# Effects of Navigated rTMS on Post-Stroke Upper-Limb Function: A Systematic Review and Meta-Analysis of Randomized Controlled Trials

**DOI:** 10.3390/brainsci15111247

**Published:** 2025-11-20

**Authors:** Jungwoo Shim, Changju Kim

**Affiliations:** 1 Department of Rehabilitation Medicine, Chungnam National University Sejong Hospital, Sejong-si 30099, Republic of Korea; sjw0812@naver.com; 2 Department of Physical Therapy, Cheongju University, Cheongju-si 28497, Republic of Korea

**Keywords:** repetitive transcranial magnetic stimulation, navigated rTMS, stroke, meta-analysis

## Abstract

**Objectives:** Neuronavigation may improve the precision and reproducibility of repetitive transcranial magnetic stimulation (rTMS) by aligning stimulation with individualized targets. Whether navigation-guided rTMS benefits post-stroke upper-limb recovery is unclear. We conducted a PRISMA-compliant systematic review and meta-analysis to estimate the effect of navigated rTMS, added to standard rehabilitation, versus sham. **Methods:** The protocol was registered in PROSPERO (CRD420251165052). Two reviewers independently searched CENTRAL, MEDLINE, Embase, CINAHL, Web of Science, and Google Scholar (October 2025), screened records, extracted data, and assessed risk of bias (Cochrane RoB-1). The prespecified primary endpoint was changed in Fugl–Meyer Assessment of the upper extremity (FMA-UE) from baseline to end of treatment. Effects were pooled as mean differences under random-effects models. When change-score standard deviations (SDs) were unavailable, they were derived from pre/post SDs assuming within-person correlation *r* = 0.5; sensitivity analyses used *r* = 0.7 and *r* = 0.9. Multi-arm trials were combined to avoid double counting. **Results:** four randomized, sham-controlled trials (*n* = 297) contributed end-of-treatment change in FMA-UE. The pooled effect favored navigated rTMS but was not statistically significant (MD 3.65, 95% CI −1.84 to 9.13; I^2^ = 73%). Sensitivity analyses with higher r produced directionally consistent estimates. A subgroup of 2-week (10-session) protocols (*k* = 3) showed a significant benefit (MD 7.09, 95% CI 4.14 to 10.05; I^2^ = 0%). Most risk-of-bias domains were low risk. **Conclusions:** Navigated rTMS did not show a consistent short-term advantage over sham on FMA-UE across heterogeneous protocols. A positive signal in standardized 2-week courses supports further adequately powered multicenter randomized controlled trials (RCTs) with harmonized protocols and complete variance reporting.

## 1. Introduction

Post-stroke upper-limb paresis affects more than 70% of survivors, and over 60% experience persistent hand dexterity deficits [1]. Although standard rehabilitation, comprising occupational and physical therapy, facilitates neuroplastic reorganization and functional recovery, many patients plateau after an initial improvement phase, leaving substantial residual disability [2,3]. As an adjunct to address these limitations, repetitive transcranial magnetic stimulation (rTMS) has been widely investigated to modulate cortical excitability and promote motor relearning [4].

rTMS typically targets the contralesional primary motor cortex (M1) with low-frequency (1 Hz) inhibitory stimulation to downregulate maladaptive interhemispheric inhibition [5,6,7], or the ipsilesional M1 with high-frequency (≥10 Hz) or intermittent theta-burst (iTBS) excitatory stimulation to enhance corticospinal output and motor relearning [8,9]. However, conventional scalp-based targeting (e.g., the 10–20 system) is prone to inter-individual and inter-session localization errors, which can compromise stimulation precision and reproducibility [10].

Neuronavigation was introduced to address targeting imprecision by using structural images (for example, magnetic resonance imaging [MRI]) or functional cues (for example, functional near-infrared spectroscopy [fNIRS] activation maps and MEP-defined hotspots) to guide coil placement in real time, thereby increasing stimulation accuracy, reducing off-target effects, and facilitating protocol standardization across sites [11,12,13]. Nevertheless, randomized controlled evidence that evaluates the clinical efficacy of navigated rTMS remains limited, and the findings to date are inconsistent [14]. Although rTMS has been applied in multiple indications, including depression, aphasia, anxiety disorders, pain, and tinnitus, trials focused on post-stroke upper-limb motor recovery are comparatively scarce [15,16,17,18,19,20]. In a large multicenter, double-blind randomized trial, navigated rTMS did not demonstrate superiority over sham at longer follow-up after completion of treatment [14], whereas smaller single-center trials suggest that MRI-guided low- or high-frequency rTMS or fNIRS-guided iTBS may yield improvements in upper-limb function at end of treatment (EOT) [21,22,23]. In addition, even among navigated studies, substantial methodological heterogeneity, including stimulation polarity and target (contralesional inhibition vs. ipsilesional facilitation), session number and pulse dose, and the intensity of concomitant rehabilitation, complicates direct comparison and quantitative synthesis [14,21,22,23].

Despite the technical advantages of neuronavigated TMS, several methodological and practical limitations remain. The use of nTMS requires expensive equipment, complex setup procedures, and trained operators, which may restrict its accessibility in typical rehabilitation settings. Moreover, the absence of standardized navigation protocols and variability in targeting accuracy across devices can lead to inconsistent stimulation results between studies [24]. Addressing these challenges will be essential for improving reproducibility and facilitating the broader clinical adoption of navigated TMS in stroke rehabilitation research.

To objectively evaluate these heterogeneous findings, the Fugl–Meyer Assessment of the upper extremity (FMA-UE) has been widely adopted as a standardized and validated measure of post-stroke motor recovery [25]. It quantitatively assesses reflex activity, synergistic movement patterns, and isolated joint control, providing a consistent index of therapeutic efficacy in upper-limb rehabilitation trials.

Several systematic reviews and meta-analyses have examined the efficacy of rTMS in various clinical populations, including stroke, depression, and Parkinson’s disease [4,26,27]. Previous reviews have mainly focused on conventional, non-navigated stimulation protocols, reporting heterogeneous and modest therapeutic effects. However, none of these reviews have specifically addressed neuronavigated rTMS or compared its methodological advantages over non-navigated approaches. This evidence gap forms the rationale for the present review.

Against this background, we conducted a systematic review and meta-analysis to synthesize evidence from randomized controlled trials examining whether image- or function-guided (navigated) rTMS, when added to standard rehabilitation, enhances post-stroke upper-limb outcomes. We prespecified the FMA-UE as the common clinical endpoint and conducted qualitative and quantitative analyses to estimate pooled effects and characterize heterogeneity.

The remainder of this paper is structured as follows: Section 2 describes the search strategy, inclusion criteria, and analytical methods; Section 3 presents the meta-analytic results; and Section 4 discusses the findings, methodological implications, and future research directions.

## 2. Materials and Methods

### 2.1. Study Design

This work is a systematic review and meta-analysis that synthesizes the effects of navigated rTMS on upper-limb outcomes in patients with stroke, using both qualitative and quantitative approaches. The review was conducted in accordance with the Preferred Reporting Items for Systematic Reviews and Meta-Analyses (PRISMA) guidelines [28]. The protocol was prospectively registered in the International Prospective Register of Systematic Reviews (PROSPERO) [29] under the identifier CRD420251165052.

### 2.2. Eligibility Criteria

Eligibility criteria were framed using the PICOSD framework (Participants, Intervention, Comparison, Outcomes, Study design) [30].

#### 2.2.1. Inclusion Criteria

Participants (P). Adults with stroke (ischemic or hemorrhagic), any lesion side, any chronicity, presenting upper-limb motor impairment.

Intervention (I). Navigated rTMS targeting the motor network (for example, MRI-guided or function-guided such as fNIRS- or MEP-based localization), delivered as an adjunct to standard rehabilitation.

Comparison (C). Sham rTMS with otherwise comparable rehabilitation. Trials with additional co-interventions were eligible if co-interventions were balanced across groups.

Outcomes (O). Upper-limb motor function measured by the FMA-UE (0–66) reported directly as post-treatment values or as change from baseline, or derivable from subscales and timepoints specified in the trial. End-of-treatment outcomes were prespecified as primary.

Study design (SD). Randomized, double- or single-blind, sham-controlled trials published as full-text articles.

#### 2.2.2. Exclusion Criteria

We excluded randomized trials that did not include a navigated rTMS intervention, non-human or preclinical studies, non-English publications, and conference abstracts, posters, or proceedings without a peer-reviewed full text.

### 2.3. Search Strategy

The studies included in this review were searched in October 2025 by two reviewers with experience in meta-analysis, independently of each other. The search strings were constructed by combining terms representing P, I, and SD, and the Medical Subject Headings (MeSH) were consulted. International electronic databases were systematically searched, including Cochrane Central Register of Controlled Trials (CENTRAL), Cumulative Index of Nursing and Allied Health Literature (CINAHL), Excerpta Medica Database (Embase), Medical Literature Analysis and Retrieval System Online (MEDLINE), Web of Science, and Google Scholar.

The search used the following combination of keywords and index terms: (stroke OR poststroke OR hemiparesis OR hemiplegia) AND (repetitive transcranial magnetic stimulation OR rTMS OR theta-burst stimulation OR iTBS) AND (navigat OR neuronavigat OR image-guided OR function-guided OR MRI-guided OR fNIRS-guided) AND (random OR randomized controlled trial OR sham-controlled OR double blind OR single blind).

The search strings were enclosed in quotation marks (“ ”) to ensure accurate phrase matching. Boolean operators (AND, OR) were used to combine terms, and searches were conducted within the title, abstract, and keyword fields of each database to enhance relevance.

Scopus was not included because its coverage substantially overlaps with Web of Science and Embase, particularly in biomedical and rehabilitation literature [31]. Including all three highly overlapping databases would have markedly increased the number of duplicate records without improving search sensitivity. The final selection of databases—CENTRAL, CINAHL, Embase, MEDLINE, Web of Science, and Google Scholar—provided comprehensive and high-quality coverage of relevant studies addressing our specific research question.

Although only four studies met the final inclusion criteria, this reflects the stringent methodological standards applied—specifically the inclusion of high-quality randomized controlled trials (RCTs) incorporating navigated or guided rTMS for post-stroke rehabilitation—rather than a limitation of the search strategy. The comprehensive search confirms that, at present, relatively few high-quality trials on this specific topic have been published.

### 2.4. Data Extraction

Records retrieved from the aforementioned electronic databases were exported and de-duplicated in Microsoft Excel for Microsoft 365 (Microsoft, Redmond, WA, USA). In accordance with PRISMA recommendations, two reviewers screened titles and abstracts, after which full texts of potentially eligible studies were assessed. Any discrepancies were resolved through joint appraisal of the full text and consensus, and studies meeting all criteria were included in the final analysis.

### 2.5. Quality Assessment

For randomized controlled trials, risk of bias was assessed using the seven-domain tool developed by the Cochrane Bias Methods Group. Two reviewers independently judged each domain as low risk (+), high risk (−), or unclear (?), following the criteria described in the Cochrane Handbook.

Low risk (+) was assigned when all methodological criteria such as adequate random sequence generation, allocation concealment, blinding, complete outcome data, and absence of selective reporting were sufficiently met. High risk (−) was assigned when clear methodological flaws were identified that could bias the results. Unclear (?) indicated that insufficient information was provided to permit a definitive judgment.

Disagreements were resolved by re-examination of the full text and consensus; when necessary, a third reviewer was consulted [32].

### 2.6. Strategy for Data Synthesis

Data synthesis was conducted using software designed for systematic reviews (RevMan version 5.4; The Cochrane Collaboration, London, UK). When studies reported a common variable suitable for pooling, or when quantitatively comparable pre- and post-intervention measures were available, a meta-analysis was performed. Meta-analysis was undertaken only when three or more studies contributed data to a given outcome.

For continuous outcomes measured on the same scale, we used the mean difference (MD) and applied a random-effects model to account for between-study heterogeneity [33]. Statistical heterogeneity was assessed with Cochran’s Q (chi-squared) test and the I^2^ statistic; I^2^ values of ≥75% were interpreted as high heterogeneity and <40% as low heterogeneity [34]. Potential publication bias was explored using funnel plots in RevMan [35].

#### 2.6.1. Sensitivity Analyses

We prespecified sensitivity analyses to examine robustness to analytic assumptions: (i) varying the within-person pre–post correlation (*r*) used to derive change-score standard deviations (SDs) (*r* = 0.5 primary; *r* = 0.7 and *r* = 0.9 in sensitivity analyses), (ii) excluding studies requiring graph-based extraction or imputation, and (iii) leave-one-out analyses. Where appropriate, we additionally report Hartung–Knapp adjustments and prediction intervals in Supplementary Materials.

#### 2.6.2. Computation of Change Scores and Variances

The primary effect measure was the mean difference in change (post−pre). When studies did not report the SD of change, it was derived from pre- and post-treatment SDs assuming a within-person correlation (*r*), as shown in Equation (1):

(1) $${SD}_{∆}=\sqrt{SD_{pre}^{2}+SD_{post}^{2}-2rSD_{pre}SD_{post}}$$

For trials reporting arm-level means and 95% confidence intervals (CIs) or paired *t* statistics, SD of change was back-calculated using standard formulas. For multi-arm trials, active arms were combined to avoid double-counting the control group, using Cochrane-recommended formulas for pooled means and SDs [36]. When FMA-UE was reported only as subscales, total scores (0–66) were reconstructed, and subscale SDs were conservatively combined under an independence assumption. Sensitivity analyses were also conducted to assess the robustness of these assumptions.

## 3. Results

### 3.1. Literature Search and Characteristics of the Included Randomized Clinical Trials

**Figure 1:**
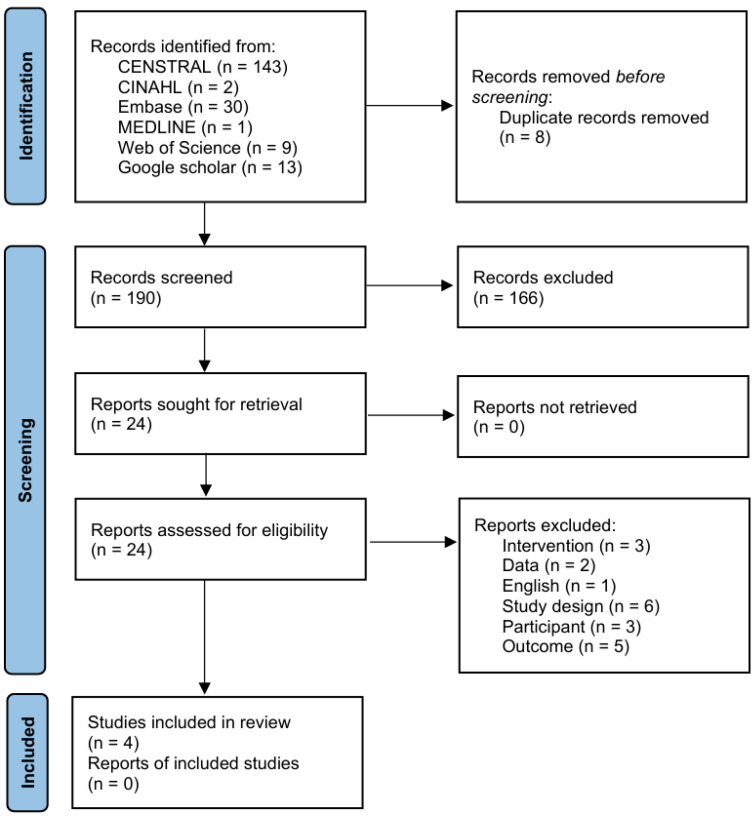
PRISMA flow diagram.

A total of 198 records were identified through international databases. Eight duplicates were removed in Microsoft Excel, and 166 records were excluded after title/abstract screening. Of the 24 full texts assessed, 20 were excluded for the following reasons: different intervention (*n* = 3), no extractable data (*n* = 2), non-English publication (*n* = 1), inappropriate study design (*n* = 6), ineligible participants (*n* = 3), and outcomes not meeting eligibility (*n* = 5). Ultimately, four RCTs met the inclusion criteria and were incorporated into both the qualitative and quantitative syntheses [14,21,22,23] (Figure 1).

During full-text screening, the E-FIT trial [37] was identified as a follow-up study of the NICHE trial [14]. Although E-FIT utilized an improved inert sham coil, it was likely conducted with the same participant cohort from the NICHE active arm. Consequently, the E-FIT trial was excluded from the quantitative synthesis to avoid data redundancy and maintain sample independence, a critical methodological consideration for preventing overestimation of the pooled effect size.

### 3.2. Methodological Quality Assessment

**Figure 2:**
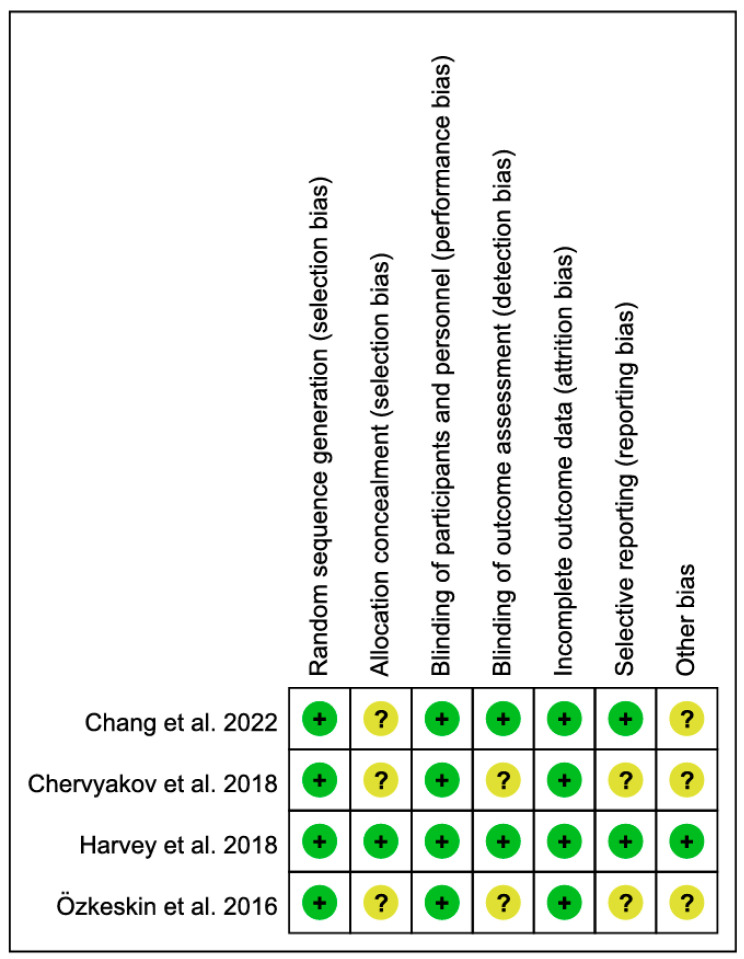
Risk of bias summary: review of authors’ judgments about each item for each included study Harvey et al., 2018, Chervyakov et al., 2018, Özkeskin et al., 2018, Chang et al., 2022 [14,21,22,23].

Inter-rater agreement across the four RCTs was 100%. Using the seven-domain Cochrane RoB tool, the judgments were as follows: random sequence generation (+: 4), allocation concealment (+: 1, ?: 3), blinding of participants and personnel (+: 4), blinding of outcome assessment (+: 2, ?: 2), incomplete outcome data (+: 4), selective reporting (+: 2, ?: 2), and other bias (+: 1, ?: 3). Overall, most domains were low risk, with residual uncertainty concentrated in allocation concealment, outcome-assessor blinding, selective reporting, and other bias (Figure 2).

### 3.3. Navigated rTMS for Patients with Stroke

Across the four included RCTs, a total of 297 patients with stroke were enrolled. Interventions used MRI-based neuronavigation or fNIRS-based functional guidance to localize the target, followed by either low-frequency (1 Hz) inhibitory or high-frequency (≥10 Hz)/iTBS excitatory rTMS protocols. The treatment duration was 2 weeks (10 sessions) in three studies and 6 weeks (18 sessions) in one study. The primary outcome was upper-limb motor function assessed with the FMA-UE (Table 1).

### 3.4. Effect of Navigated rTMS on Upper Limb Function

#### 3.4.1. Primary Analysis

**Figure 3:**
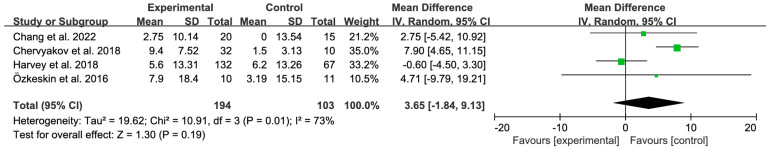
Forest plot of change in Fugl-Meyer assessment of the upper extremity at end of treatment: navigated repetitive transcranial magnetic stimulation versus sham repetitive transcranial magnetic stimulation Harvey et al., 2018, Chervyakov et al., 2018, Özkeskin et al., 2018, Chang et al., 2022 [14,21,22,23].

Four RCTs contributed change in FMA-UE (post − pre) at end of treatment [14,21,22,23] (Figure 3). Using a random-effects inverse-variance model, the pooled MD favored navigated rTMS by 3.65 points on the 0–66 FMA-UE scale (95% CI −1.84 to 9.13), which was not statistically significant (overall *Z* = 1.30, *p* = 0.19). Between-study heterogeneity was substantial (*τ*^2^ = 19.62; *χ*^2^ = 10.91, df = 3, *p* = 0.01; I^2^ = 73%), reflecting variability in stimulation polarity and site, dose, and co-interventions. For trials that did not report the SD of change, we derived it from pre- and post-treatment SDs assuming a within-person pre–post correlation of *r* = 0.5 (primary analysis).

#### 3.4.2. Sensitivity Analyses

**Figure 4:**
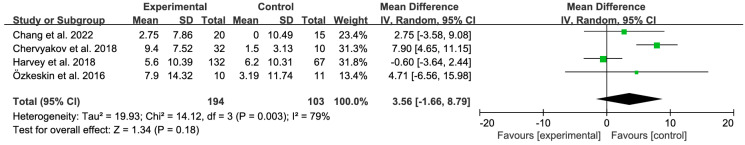
Sensitivity analysis of the pooled effect for the Fugl–Meyer Assessment of the upper extremity using an assumed within-person correlation of *r* = 0.7 for the calculation of change score standard deviations. The forest plot shows the mean difference and 95% confidence intervals for navigated repetitive transcranial magnetic stimulation versus sham stimulation across included randomized controlled trials Harvey et al., 2018, Chervyakov et al., 2018, Özkeskin et al., 2018, Chang et al., 2022 [14,21,22,23].

**Figure 5:**
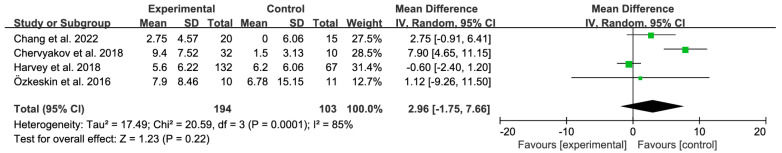
Sensitivity analysis of the pooled effect for the Fugl–Meyer Assessment of the upper extremity assuming a higher within-person correlation of *r* = 0.9. The forest plot displays the recalculated mean difference and 95% confidence intervals for navigated repetitive transcranial magnetic stimulation compared with sham stimulation, indicating the robustness of the overall results to varying correlation assumptions Harvey et al., 2018, Chervyakov et al., 2018, Özkeskin et al., 2018, Chang et al., 2022 [14,21,22,23].

To examine robustness to the correlation assumption used to derive change-score SDs, we repeated the meta-analysis with higher within-person pre–post correlations. At *r* = 0.7, the pooled MD was 3.56 points (95% CI −1.66 to 8.79; *Z* = 1.34, *p* = 0.18), with heterogeneity *τ*^2^ = 19.93; *χ*^2^ = 14.12 (df = 3), *p* = 0.003; I^2^ = 79% (Figure 4). At *r* = 0.9, the pooled MD was 2.96 points (95% CI −1.75 to 7.66; *Z* = 1.23, *p* = 0.22), with heterogeneity *τ*^2^ = 17.49; *χ*^2^ = 20.59 (df = 3), *p* < 0.001; I^2^ = 85% (Figure 5). In both re-analyses, the direction of effect favored navigated rTMS but statistical significance was not achieved and heterogeneity remained substantial. As r increased (thereby reducing the derived SD of change), confidence intervals for studies requiring derivation (Chang et al., 2022 [23], Harvey et al., 2018 [14], Özkeskin et al., 2016 [22]) narrowed modestly and their weights increased, whereas the study with SD obtained from test statistics (Chervyakov et al., 2018 [21]) was unchanged.

#### 3.4.3. Subgroup Analysis by Treatment Duration (2-Week Protocols)

**Figure 6:**
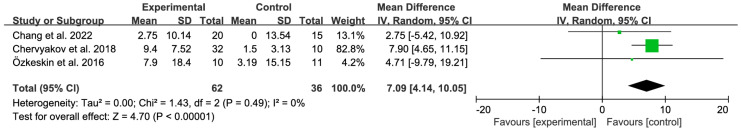
Forest plot for the 2-week (10-session) subgroup: navigated repetitive transcranial magnetic stimulation vs. sham repetitive transcranial magnetic stimulation on change in Fugl-Meyer assessment of the upper extremity at end of treatment Chervyakov et al., 2018, Özkeskin et al., 2018, Chang et al., 2022 [21,22,23].

Restricting the synthesis to trials that delivered 10 sessions over 2 weeks [21,22,23], the pooled MD for change in FMA-UE (0–66) was 7.09 points (95% CI 4.14 to 10.05; *Z* = 4.70, *p* < 0.00001), indicating a significant benefit of navigated rTMS with negligible heterogeneity (*τ*^2^ = 0.00; *χ*^2^ = 1.43, df = 2, *p* = 0.49; I^2^ = 0%). For studies without reported SD of change, SDs were derived assuming *r* = 0.5. Given the small number of trials and the dominant weight of a single study, these findings should be interpreted as exploratory (Figure 6).

### 3.5. Publication Bias

In this review, six studies were synthesized for the systematic review and meta-analysis based on the eligibility criteria. According to Cochrane Reviews, assessment of publication bias is not recommended when fewer than 10 studies are included in the analysis [38]. Therefore, publication bias was not evaluated in this study.

## 4. Discussion

This systematic review and meta-analysis quantified the effect of neuronavigation-guided rTMS, when added to standard rehabilitation, on upper-limb function in randomized, sham-controlled trials.

In response to the predefined research question, this meta-analysis found no statistically significant pooled MD in FMA-UE at end of treatment between navigated rTMS and sham stimulation, while sensitivity and subgroup analyses provided partial support for potential benefits under standardized conditions.

In the primary analysis, there was no statistically significant difference in change in FMA-UE at end of treatment (pooled MD, 3.65 points; 95% CI, −1.84 to 9.13; I^2^ = 73%) [14,21,22,23]. These estimates were synthesized by deriving change-score standard deviations under an assumed within-person pre–post correlation of *r* = 0.5; repeating the analysis with *r* = 0.7 and *r* = 0.9 yielded directionally consistent effects with modestly narrower confidence intervals, and the overall conclusion remained unchanged. In contrast, an exploratory subgroup restricted to 2-week (10-session) protocols showed a significant benefit with negligible heterogeneity (I^2^ = 0%) [21,22,23], suggesting that harmonizing treatment duration and session number may improve the consistency of effect estimates across trials.

In context, neuronavigation-guided rTMS was introduced to reduce session-to-session localization error and to enhance reproducibility by delivering stimulation precisely to individualized targets defined by structural imaging or functional markers [12,39]. Nevertheless, findings have been inconsistent: a multicenter, double-blind randomized trial did not demonstrate superiority over sham at longer follow-up [14], whereas single-center small RCTs using MRI-guided low- or high-frequency rTMS or fNIRS-guided iTBS suggested possible improvements in upper-limb function at end [22] of treatment [21,23]. This inconsistency was also reflected in our synthesis, likely because protocols differed substantially in stimulation polarity and target (contralesional 1 Hz inhibition versus ipsilesional high-frequency or iTBS facilitation), number of sessions and pulse dose, and the intensity of concomitant rehabilitation, and because in several trials the variance of change scores was not reported and had to be derived from pre–post variances. In addition, meaningful within-group improvement was frequently observed in sham plus rehabilitation, which tended to reduce between-group contrasts.

Our findings are consistent with the heterogeneity reported in the non-navigated rTMS literature. Targeting based on scalp coordinates such as the 10–20 system is limited by interindividual anatomical variability, which can introduce variability in effective field strength and volume and thereby reduce the consistency of clinical effects [40]. Recent reviews and meta-analyses of non-navigated rTMS in post-stroke upper-limb rehabilitation suggest possible benefits, but they also repeatedly note wide dispersion of effect sizes and high between-study heterogeneity driven by differences in stimulation polarity and target, dose, and assessment time points [41,42]. While neuronavigation has clear technical potential to address these limitations, the existing randomized evidence is insufficient to generalize a short-term clinical superiority over sham [14,21,22,23]. Nevertheless, the positive signal observed in the 2-week (10-session) subgroup suggests that stricter standardization of treatment duration, session number, and stimulation polarity may yield more consistent and detectable clinical effects [21,22,23].

To advance this field, future research should pursue three main directions. First, conduct adequately powered multicenter RCTs directly comparing navigated with non-navigated rTMS under identical stimulation polarity, target, intensity, and session number. Second, establish uniform reporting standards by preregistering outcome time points, fully reporting arm-level change means and SDs, and harmonizing follow-up durations to enhance comparability across studies. Third, incorporate biomarker-based stratification, including corticospinal tract integrity, MEP status, and neuroimaging-derived functional connectivity markers, to identify responders and clarify mechanisms of treatment response.

To our knowledge, this is the first PRISMA-based meta-analysis to quantitatively synthesize only randomized, sham-controlled RCTs of neuronavigation-guided rTMS for post-stroke upper-limb function. Prior reviews and meta-analyses generally did not isolate navigation or combined nonrandomized evidence, which makes it difficult to determine the incremental value of neuronavigation [43,44,45]. In this study, we harmonized the endpoint at end of treatment (EOT) and the clinical scale (FMA-UE, 0–66), prespecified the mean difference in change scores as the primary effect measure, combined multi-arm trials to avoid double counting, and performed sensitivity analyses for the assumed pre–post correlation *r* to strengthen interpretability [14,21,22,23].

## 5. Conclusions

In conclusion, although neuronavigation can increase targeting precision and reproducibility, the current randomized evidence does not consistently demonstrate short-term superiority of navigated rTMS over sham stimulation in enhancing upper-limb motor recovery as measured by FMA-UE. However, a positive signal was observed in standardized 2-week (10-session) protocols with low heterogeneity, suggesting that harmonized treatment schedules and stimulation parameters may yield more consistent therapeutic effects.

Despite the limited number of available trials, this study provides a methodologically rigorous synthesis of current evidence, emphasizing the need for further well-designed research. Future studies should include adequately powered multicenter RCTs directly comparing navigated and non-navigated rTMS, adopt standardized outcome reporting, and integrate neurophysiological or imaging biomarkers to identify patient subgroups most likely to benefit from neuronavigation-guided stimulation.

Collectively, these directions will help clarify the incremental clinical value of neuronavigation and guide its optimal implementation in post-stroke neurorehabilitation.

## Figures and Tables

**Table 1:** Characteristics of included studies.

Study	Participants	Therapeutic Intensity	Outcome
Chang et al., 2022 [23]	EG = 20 CG = 15	2 weeks, 5 sessions/week (10 sessions); fNIRS-guided iTBS over ipsilesional M1, 80% MT, 600 pulses/session.	Upper limb function: FMA-UE, WMFT
Chervyakov et al., 2018 [21]	EG = 32 CG = 10	2 weeks, 5 sessions/week (10 sessions); MRI-guided rTMS (Nextstim) over M1 at 90% RMT, 1 Hz (ipsilesional), 10 Hz (contralesional), or dual protocol (~1200–1500 pulses/session).	Upper limb function: FMA-UE
Harvey et al., 2018 [14]	EG = 132 CG = 67	6 weeks, 3 sessions/week (18 sessions); MRI-guided rTMS 1 Hz at 90% RMT, ~1200 pulses/session + task-oriented training.	Upper limb function: FMA-UE, WMFT, ARAT Quality of life: SIS-16, EQ-5D Spasticity: MAS
ÖZKESKİN et al., 2016 [22]	EG = 10 CG = 11	2 weeks, 5 sessions/week (10 sessions); MRI-guided 1 Hz rTMS at 90% RMT, 1500 pulses/session + Brunnstrom hand training.	Upper limb function: FMA-UE, JTT

EG: experimental group; CG: control group; fNIRS: functional near-infrared spectroscopy; iTBS: intermittent theta-burst stimulation; MT: motor threshold; FMA-UE: Fugl–Meyer assessment of the upper extremity; WMFT: Wolf motor function test; MRI: magnetic resonance imaging; rTMS: repetitive transcranial magnetic stimulation; RMT: resting motor threshold; ARAT: Action research arm test; SIS-16: Stroke impact scale (16-item version); EQ-5D: EuroQol 5-dimension scale; MAS: modified Ashworth scale; JTT: Jebsen–Taylor hand function test.

## Data Availability

All data supporting the findings of this study are contained within the article and its Supplementary Materials (extraction sheets, effect-size calculations, and sensitivity analysis outputs). Any additional materials are available from the corresponding author on reasonable request.

## Supplementary Materials

The following supporting information can be downloaded at: https://www.mdpi.com/article/10.3390/brainsci15111247/s1, PRISMA 2020 Checklist.
